# Mathematical Model of HIV/AIDS Considering Sexual Preferences Under Antiretroviral Therapy, a Case Study in San Juan de Pasto, Colombia

**DOI:** 10.1089/cmb.2021.0323

**Published:** 2022-05-10

**Authors:** Cristian C. Espitia, Miguel A. Botina, Marco A. Solarte, Ivan Hernandez, Ricardo A. Riascos, João F. Meyer

**Affiliations:** ^1^ Universidad de los Llanos, Villavicencio, Colombia.; ^2^ Instituto Departamental de Salud de Nariño, Pasto, Colombia.; ^3^ Hospital Universitario Departamental, Pasto, Colombia.; ^4^ Universidad Cooperativa de Colombia, Pasto, Colombia.; ^5^ Universidad Estadual de Campinas, Instituto de Matemáticas, Estadistica Y Computación Cientifica.

**Keywords:** antiretroviral therapy, human immunodeficiency virus, system of ordinary differential equations

## Abstract

While several studies on human immunodeficiency virus (HIV)/acquired immunodeficiency syndrome (AIDS) in the homosexual and heterosexual population have demonstrated substantial advantages in controlling HIV transmission in these groups, the overall 2benefits of the models with a bisexual population and initiation of antiretroviral therapy have not had enough attention in dynamic modeling. Thus, we used a mathematical model based on studying the impacts of bisexual behavior in a global community developed in the PhD thesis work of Espitia (2021). The model is governed by a nonlinear ordinary differential equation system, the parameters of which are calibrated with data from the cumulative cases of HIV infection and AIDS reported in San Juan de Pasto in 2019. Our model estimations show which parameters are the most influential and how to modulate them to decrease the HIV infection.

## 1. INTRODUCTION

In the beginning of the 80's, the Center for Disease Control and Prevention (CDC) caught the world's attention because of its presentation of the acquired immunodeficiency syndrome (AIDS) caused by the human immunodeficiency virus (HIV). The scientific community optimistically believed that an effective vaccine would soon be developed. However, almost 40 years later, neither a vaccine nor a successful treatment could stop the transmission of this virus.

An alternative treatment called “antiretroviral therapy” (ART) is currently the best option for a long-lasting viral suppression and subsequently for a reduction of mortality. Nowadays, available drugs do not completely eradicate the HIV infection, but they decrease virus replication and consequently reduce the morbidity and mortality, which are generally associated with AIDS.

According to the United Nations Program on HIV/AIDS (UNAIDS, 2021), since the beginning of the epidemic and until 2019, 75.7 million people contracted HIV infection, and 32.7 million people died from AIDS. At the end of 2019, 38 million people were living with HIV worldwide, and 1.7 million people contracted HIV infection in that year; besides, more than 690,000 people died from AIDS-related illnesses.

In 2014, UNAIDS proposed the Fast-Track Treatment target called “90-90-90 intervention,” in which by 2020, it was required that 90% of individuals living with HIV infection worldwide should be diagnosed, 90% of these should have enrolled in ART, and 90% of individuals receiving ART should have a suppressed viral load. In Colombia in 2019, ART coverage was 85.63%, and 72.09% had undetectable viral load, <50 copies/mm^3^. The department of *Nar*iño had a coverage of 90.26% of ART with 72.97% of undetectability (CAC 2019).

One of the first investigations in HIV/AIDS mathematical modeling was carried out by Anderson et al. (1986) who presented two epidemiological deterministic mathematical models: the first one is sexual transmission in susceptible infectious people living with AIDS and noninfectious zeropositive; the second one is sexual transmission between susceptible individuals, two classes of infectious, people living with AIDS and, noninfectious zeropositive. Since then, a large number of variations and improvements have been made using current mathematical and medicinal tools. These models can help predict the future behavior of the disease and offer a better understanding of its epidemiological patterns.

The following review is a list of the main articles about HIV/AIDS from 2012 to 2019: Kaur et al. (2012), explored the spread of HIV in children, adults, and seniors taking into consideration horizontal and vertical transmissions. Sun et al. (2013) investigated the epidemic among men who have sex with men enrolled in public health programs such as condom use and ART, dividing the infected population according to CD4^+^ cell counts in the blood. Bhunu et al. (2014) studied the dynamics of prostitution in the transmission of the disease based on an epidemiological mathematical model in susceptible infected individuals living with AIDS, in prostitutes and nonprostitutes receiving ART and considering viral load in the force of infection. Bhunu (2015) claimed that the care for individuals living with HIV/AIDS is more than the provision of ART, and the homelessness on HIV/AIDS transmission is described through a deterministic mathematical model.

The PhD thesis of Afassinou (2016) introduced a compartmental mathematical model incorporating pre-exposure prophylaxis in the susceptible population and infected individuals. A research about injectable drug users was presented by Yang et al. (2017); they studied female sex workers and senior male clients with heterosexual transmission using an epidemiological deterministic model. Omondi et al. (2018) also approached the dynamics in the sexually mature age group aged 15 years in a population of susceptible and infected individuals considering CD4^+^ T cell counts and individuals enrolled in ART. Finally, Omondi et al. (2019) studied the HIV transmission dynamics using a compartmental model. They considered heterosexual transmission in two age groups in Kenya; young adults aged 15–24 and adults aged 25 and older, each population was subdivided into susceptible and infected people receiving ART treatment.

The previous literature review shows a pattern of HIV/AIDS mathematical models, which is often considered in a heterosexual population and a few times in heterosexuals with homosexuals separately. However, populations consisting of heterosexuals, bisexuals, and homosexuals at the same time are not included; it means that bisexual behavior is not studied in this mathematical modeling. Thus, we investigate the influence of sexual intercourse among these groups on the transmission of HIV and subsequent AIDS always under ART.

The proposed model is an original result of an extensive literary review of researches about the following: first, epidemiological HIV/AIDS mathematical models, second, biological researches about HIV behavior and later AIDS, and third, psychological aspects regarding homosexual and bisexual contacts. Thus, our model considers one of its main contributions the homosexual, heterosexual, and bisexual contacts with different sexual preferences. Sensibility of parameters in the model was analyzed according to the Latin Hypercube Sampling Method with the aim of knowing which ones have greater sensitiveness to the variations on the initial conditions.

## 2. BACKGROUND

A contextualization of sexual contact and its transmission mechanisms is provided in this section, it is provided a contextualization about sexual contact and its transmission mechanisms. Heterosexual and homosexual contact is contemplated as the main route of HIV/AIDS contagion, and bisexual contact in heterosexuals and homosexual individuals as another way to get infected. In this regard, the transmission between exclusively homosexual women will not be taken into account due to the absence of the exchange of body fluids, which is the principal mechanism of sexual transmission. However, there are some cases of transmission mainly due to the exchange of sex toys, for more information, see works such as Lloyd and May (1996), Mastro and De Vincenzi (1996), Kwakwa and Ghobrial (2003), and Chan et al. (2014).

Sexual contact between homosexual and heterosexual men is justified by Rosario et al. (2006) and Thompson and Morgan (2008), where the authors state that: “there exist individuals that change their sexual behavior depending on the situation or at different stages in their life. A possibly common and transient example of situational sexuality is the person who self identifies as heterosexual, but will sexually interact with a member of the same sex when lacking other opportunities. Less transient but also possibly common, a person who self identifies as homosexual or lesbian (either at the time, or later) may sexually interact with a member of the opposite sex if a same-sex relationship seems unfeasible.” As a result, in this model, sexual interaction between homosexual men and heterosexual men will be assumed.

The CDC estimates that HIV rates in men having sex with men (MSM) are higher than heterosexual contacts. In part, these differences reflect the fact that an individual MSM can engage in both insertive and receptive sexual roles (versatility), while exclusively heterosexual men and women each engage in only one of these roles (Beloqui, 2008; Simon et al., 2008; Glick et al., 2012).

## 3. METHODS

A compartmental model of HIV/AIDS is represented by a system of ordinary differential equations (ODE) formed by observing the flow of individuals from one compartment to the others. As an example, the target population is divided into three compartments, where the number of individuals changes with respect to time; the susceptible class S(t) comprises individuals who were not exposed to infection, the infected class I(t) consists of infected individuals who infect others, and the class of individuals living with AIDS A(t) involves individuals who have already developed the syndrome as a result of HIV infection. The infection occurs due to the interaction of susceptible individuals with infected ones, and depends on the initial hypothesis of the model.

The presented model was made under the advice of the HIV/AIDS infectious disease specialist Dr. Alexandre Naime Barbosa, who evaluated the dynamics hypotheses; this model is part of the doctoral thesis work in applied mathematics at the Institute of Mathematics, Statistics and Scientific Computing in State University of Campinas by Espitia (2021). The model assumed that the only way to transmit the HIV virus is through sexual relationships between heterosexuals, homosexuals, and bisexual individuals; moreover, the effect of ART among infected people is studied making use of a deterministic mathematical model of nonlinear ODE.

The total population is divided in homosexual men and heterosexual men and women; therefore, into eight classes as described in Table 1.

**Table 1.** Description of State Variables in the Model

Population	Description
*S_h_*(*t*)	Susceptible homosexual men
*I_h_*(*t*)	Untreated infected homosexual men
*S_w_*(*t*)	Susceptible women
*I_w_*(*t*)	Untreated infected women
*S_m_*(*t*)	Susceptible heterosexual men
*I_m_*(*t*)	Untreated infected heterosexual men
*T(t)*	Treated with antiretrovirals
*A*(*t*)	People living with AIDS

AIDS, acquired immunodeficiency syndrome.

**FIG. 1.**
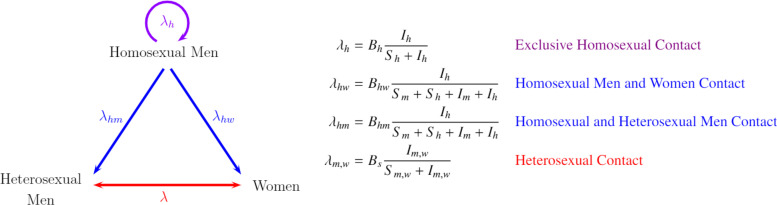
Triangle transmission between homosexual men, heterosexual men, and women.

Figure 1 represents the transmission dynamics between the three studied sexual preferences. Each vertex of the triangle represents one population, and the sides of the triangle denote the different forms of transmission between the populations involved. To begin, the exclusive transmission among homosexual men is illustrated by the upper circular arrow in purple labeled $\lambda_{h}$. Then, the transmission between homosexual and heterosexual men and the transmission between homosexual men and women are represented by blue lines labeled $\lambda_{hm}$ and $\lambda_{hw}$, respectively. Finally, heterosexual transmission between men and women is in the red line represented by λ. The direction of the arrows represents the sense of the analyzed contagion; nonetheless, contagions can biologically occur in all directions, in this work, we assume only those directions shown in Figure 1, because with the acceptance of all the directions, besides demanding a cumbersome mathematical treatment also causing the model to bring about qualitatively different equilibrium dynamics.

Consequently, it is assumed that the only form of contagion among homosexuals is among themselves, and that heterosexual people become infected due to contact with homosexual men or heterosexuals of the opposite sex. Thus, the blue lines have only one direction, while the red line between heterosexual men and women has two.

### 3.1. Mathematical model

To be able to model these contacts, the infection forces of which are shown in Figure 1, these mathematical expressions must be able to represent the way in which susceptible individuals are becoming infected. $B_{h,hw,hm,s}$ represents the product between the probability of contagion $\beta_{h,hw,hm,s}$ and the rate of the number of sexual partners, $c_{h,hw,hm,s}$. In these infection forces, it was assumed that individuals under treatment T(t) and individuals living with AIDS A(t) under treatment are the least infectious because, due to ART, their viral load is undetectable, and therefore, the contagion is almost null.

#### 3.1.1. Hypotheses assumed for modeling

**H1** Constant recruitment in all susceptible classes is assumed. **H2** Sexual transmission in discordant couples is considered. **H3** Homosexual individuals get infected among themselves. The HIV transmission in a female population occurs through sexual relationships with infected heterosexual men or through sexual contact with infected homosexual men. Susceptible heterosexual men can get infected by infected women or infected homosexual men. **H4** No gender difference is taken into account for treated individuals as well as for individuals living with AIDS. **H5** Individuals living with AIDS could be treated or untreated, noting that an individual who developed AIDS during a hospital treatment will be diagnosed and enrolled in ART. **H6** Both natural mortality in all classes and induced mortality for individuals living with AIDS are considered.

The parameters used in this model are as follows: Ψ represents constant recruitment in susceptible individuals. θ is the proportion of men, while γ is that of heterosexual men. Thus, recruitments in susceptible populations of homosexual men, women, and heterosexual men are Ψθ(1−γ), Ψ(1−θ), and Ψθγ, respectively. *p* is the proportion of initially treated individuals. μ denotes the natural mortality rate. *d* is the parameter for induced disease mortality rate. δ represents the AIDS development rate in treated individuals due to ART resistance or treatment failure. α is the departure rate from all compartments of infected individuals. $\beta_{h,hw,hm,s}$ means probability of sexual transmission and $c_{h,hw,hm,s}$ denotes sexual partner rate. The diagram and ODE system is shown in Eq. (2), and its dynamics is governed by a nonlinear and autonomous system of ODE represented in Eq. (2).

**FIG. 2.**
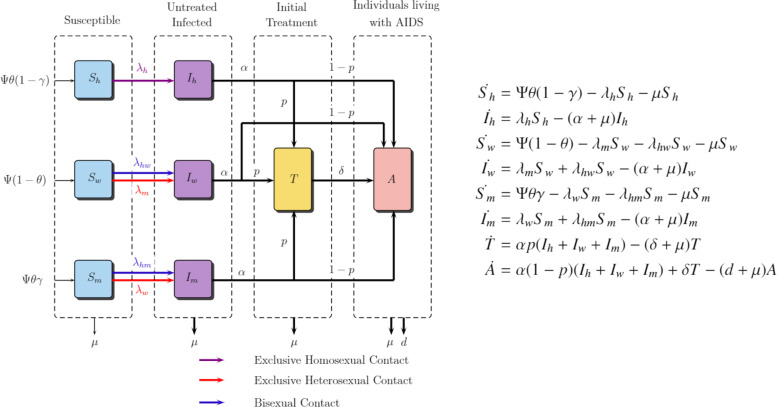
Diagram and ODE system for triangle transmission model. ODE, ordinary differential equations.

The basic reproduction number, denoted by *R*_0_, represents the expected number of secondary cases produced by a typically infected individual in a completely susceptible population. If $R_{0}<1$, then one infected individual can generate less than one new infection over the course of the infectious period; therefore, the disease cannot grow. However, if $R_{0}>1$, then one infected individual produces more than one new infection, and thus, the disease invades the population and it is considered an epidemic. For important features of *R*_0_, we recommend Holland (2007). The next-generation method exposed in Van den Driessche (2017) is used, the results of which indicate that this basic reproduction number for the ODE model presented in Figure 2 is given by the following:

(1) $$R_{0}=\max\left\{\frac{c_{h}\beta_{h}}{\alpha +\mu },\frac{c_{s}\beta_{s}}{\alpha +\mu }\right\}=\max\left\{R_{0}^{hom},R_{0}^{het}\right\}$$

**FIG. 3.**
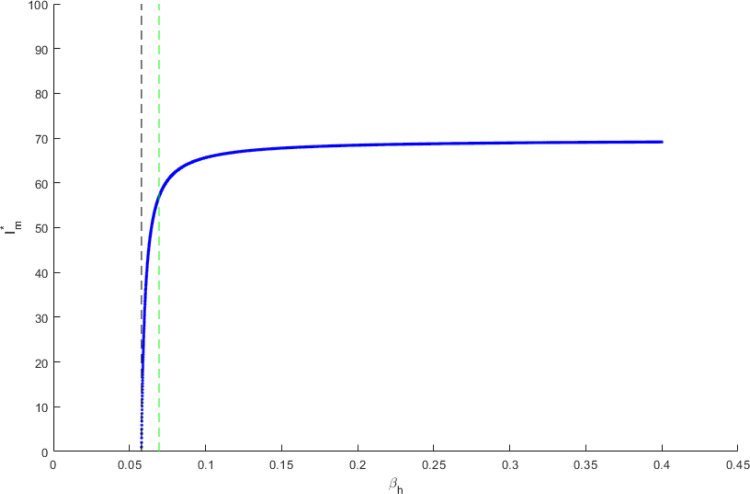
Bifurcation diagram of untreated infected heterosexual men, in function of transmission probability in homosexual contact.

A forward bifurcation is present in this model as shown in the diagram of Figure 3. Two dotted vertical lines have been illustrated; the black one represents $R_{0}^{hom}=1$, while the green one marks $R_{0}^{het}=1$.

Local and global stability of stationary points was demonstrated in the PhD thesis work (Espitia, 2021). Thus, we claim that stability does not depend on initial conditions.

## 4. DATA AND PARAMETERS FOR HIV/AIDS MODEL IN SAN JUAN DE PASTO

Data adjustment for HIV infection in San Juan de Pasto was made using government bibliographical sources such as data from the Departmental Health Institute of *Nar*iño (IDSN, 2019) and the National Administrative Department of Statistics (DANE, 2019), as well as scientific references. Table 2 summarizes active cases in San Juan de Pasto from 1989 to 2019.

**Table 2.** Active Cases Reported in San Juan de Pasto from 1989 to 2019

Year	1989	1990	1991	1992	1993	1994	1995	1996	1997	1998	1999
Individuals	1	2	3	6	3	16	11	8	8	21	21
Year	2000	2001	2002	2003	2004	2005	2006	2007	2008	2009	2010
Individuals	24	25	41	44	50	54	75	67	35	36	35
Year	2011	2012	2013	2014	2015	2016	2017	2018	2019		Total
Individuals	30	39	61	54	73	67	101	101	121		1233

Source: Departmental Institute of Health of Nariño.

With the aim of knowing the dynamical behavior and the effects of varying the parameters in the ODE system, we carry out numerical simulation following Runge-Kutta's mathematical method of order 4 determining the baseline values of parameters known from the literature that correspond to available experimental data and biological facts. The unknown parameters were estimated intuitively based on the general behavior of the country and department data. The parameter values are approximated as follows: Ψ, which is defined as the recruitment in the susceptible population, is estimated using data from the National Census of Population and Housing (CNPV 2019); with regard to San Juan de Pasto, this value is given by 333 individuals per year. The natural death rate, μ, is estimated by DANE (2019) and based on life expectancy, which is reported as 77.3 years of age, and thus, $\mu =\frac{1}{77.3}\approx 0.0129$. The AIDS-related death rate, *d*, based on the life expectancy of individuals living with AIDS, as stated by Nettleman (2019), is on average 3 years, and therefore, $d=\frac{1}{3}\approx 0.3333$.

Sources such as Fernández (2019) and Choi et al. (2020) were useful in estimating parameters such as sexual partners, $c_{h,s,hw,hm}$. ART parameters were adapted from CAC (2019). All parameter values are listed in Table 3, and the initial conditions were assumed by DANE (2019) and UNAIDS (2021); those are shown in equation (2).

**Table 3.** Parameters of the Human Immunodeficiency Virus/Acquired Immunodeficiency Syndrome Model for Case Study in San Juan de Pasto

Parameters	Value	Unit	Source
Ψ	333	Individuals/year	CNPV (2019)
*θ*	0.48	Nondimension	DANE (2019)
*γ*	0.92	Nondimension	DANE (2019), Williams (2019)
*p*	0.90	Nondimension	CAC (2019), Cornejo et al. (2020)
*μ*	0.0129	1/year	DANE (2019)
*d*	0.3333	1/year	Nettleman (2019)
*δ*	0.018	1/year	Apenteng and Ismail (2019)
*α*	0.3333	1/year	Akudibillah et al. (2018)
*β_s_*	0.02	Nondimension	Ostadzad et al. (2016)
*β_h_*	0.44	Nondimension	UNAIDS (2021)
*β_hw_*	0.018	Nondimension	Assumed
*β_hm_*	0.25	Nondimension	Assumed
*c_s_*	4	1/year	Fernández (2019)
*c_h_*	7	1/year	Fernández (2019)
*C_hw_*	2	1/year	Fernández (2019)
*C_hm_*	1	1/year	Fernández (2019)

(2) $$\begin{array}{cccccccc} S_{h}\left(0\right) & =2446, & I_{h}\left(0\right) & =79, & S_{w}\left(0\right) & =189994, & I_{w}\left(0\right) & =6,\\ S_{m}\left(0\right) & =171173, & I_{m}\left(0\right) & =29, & T\left(0\right) & =107, & A\left(0\right) & =47. \end{array}$$

## 5. RESULTS

We present one main scenario and three possible additional scenarios, which, in addition to allowing a better understanding of the dynamics of HIV infection, are a fundamental tool in the design and analysis of public health policies. A common behavior in all scenarios is the decline of susceptible populations due to infected people, and thus, we only present changes in infected populations.

### 5.1. Scenarios

#### 5.1.1. Main scenario

**FIG. 4.**
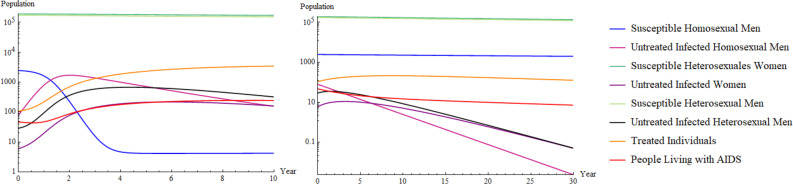
On the left, the main scenario using parameters in table description of parameters. On the right, disease-free equilibrium when the basic reproduction number in homosexuals is less than one, both in logarithm scale.

The parameter values in Table 3 correspond to a basic reproduction number in homosexuals $R_{0}^{hom}=8.89659$ and a basic reproduction number in heterosexuals $R_{0}^{het}=0.23108$. Figure 4 is a numerical simulation of populations for a period of 10 years, noticing that susceptible populations decline over time.

The disease-free equilibrium is defined as the point at which no disease is present in the population, which is generally represented in epidemiological models when the basic reproduction number is less than one. Getting to this point means controlling the epidemic and eradicating the infection in the long term, which is the main goal of every epidemiological model. As a result, the right side of Figure 4 shows the infected population when the parameter of homosexual partners is $c_{h}=0$, and thus, $R_{0}^{hom}=0<1$; it follows that the population reaches a disease-free equilibrium, and, as a consequence, the infected population is eradicated.

#### 5.1.2. Scenario 1: modifying the basic reproduction number in heterosexuals, $R_{0}^{het}$

**FIG. 5.**
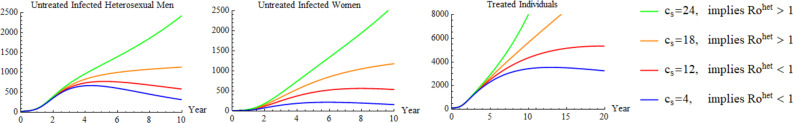
Scenario 1: modifying heterosexual partners’ rate.

We simulated the effect of considering several heterosexual partners, *c_s_* (keeping the other parameters as in Table 3), and thus, $R_{0}^{het}$ is modified. The first modification is when $R_{0}^{het}<1$, in this way $c_{s}=4$ and $c_{s}=12$ are assumed, noting that the infected heterosexual men population declines in approximately 4.5 years, and the infected women population declines in about 6 years, and thus heterosexual men decrease faster than infected women. The second modification is when $c_{s}=18$ or 24; in this case we have $R_{0}^{het}>1$, verifying that the populations of infected heterosexual men, women, and treated individuals grow over time. Figure 5 depicts this behavior.

#### 5.1.3. Scenario 2: modifying the departure rate of infected individuals α

**FIG. 6.**
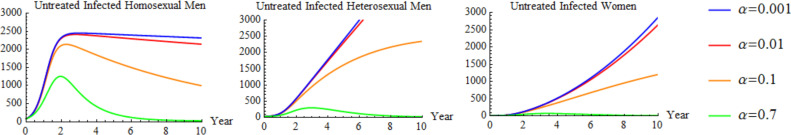
Scenario 2: modifying departure rate from infected individuals.

The departure rate of infected individuals has the greatest variability of populations; the α parameter is the most sensitive* one for the model. Susceptible populations decline, while infected individuals, treated populations, and people living with AIDS have changes, as shown in Figure 6.

#### 5.1.4. Scenario 3: modifying sexual partners between homosexual men with women, and homosexual men with heterosexual men

**FIG. 7.**
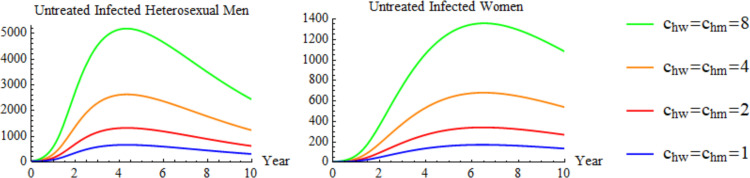
Scenario 3: modifying bisexual partner.

Figure 7 shows the effect of modifying sexual partners in a bisexual contact for infected heterosexual men and women.

## 6. DISCUSSIONS

The dynamics of the HIV/AIDS epidemic, to a large extent, depends on changes of the basic reproduction number for homosexuals, $R_{0}^{hom}$. This was also evidenced by modifying several parameters in the scenarios above. The basic reproduction number in heterosexuals, $R_{0}^{het}$, is less influential because in the Background section, it was mentioned that the probability of HIV infection in homosexuals is higher than in heterosexuals. In addition, investigations such as Cohen et al. (2011) and Del Romero et al. (2016) permit to conclude that the number of sexual partners of homosexuals is larger than the heterosexuals, thus, $R_{0}^{hom}>R_{0}^{het}$. It suggests that HIV infection can be controlled or eliminated from the community if control programs are directed toward reducing $R_{0}^{hom}$ to values less than one. The model showed the persistence of the disease when $R_{0}^{hom}>1$.

The dynamics of HIV/AIDS is, in general, too complex to allow intuitive predictions, and requires both the support of mathematical modeling for quantitative assessments and the understanding of the system functioning; furthermore, one of the most difficult tasks of mathematical modeling was the model parameters. Moreover, the proposed HIV/AIDS model tries to be as approximate as possible using real parameter values for the behavior in San Juan de Pasto. The emphasis is not on the accuracy of the scenarios, but on the actions that can be taken now as a result of seeing the state of the epidemic in the future; these actions can involve, among other things, the prevention of new infections, provision of ART, and educational campaigns such as the reduction of the number of sexual partners or use of condom for self-protection.

The model presented in this work should be treated with circumspection due to the assumptions made surrounding the estimation of the model parameters. Nonetheless, the model provides useful insights about the dynamics of the epidemic.

## 7. CONCLUSIONS

A deterministic mathematical model of nonlinear differential equations was performed to model the dynamics of HIV/AIDS in an adult population considering sexual preferences such as the following: exclusive homosexual men contact, contact between homosexual men and women, contact between homosexual and heterosexual men, and finally, heterosexual contact, according to scientific references that validate these interactions. Thus, we consider three susceptible populations: homosexual men, women and heterosexual men, and consequently infected individuals under ART and people living with AIDS.

HIV/AIDS epidemic data were collected from the Departmental Health Institute of *Nar*iño (IDSN, 2019) and the National Administrative Department of Statistics (DANE, 2019) to model HIV infection in San Juan de Pasto, Colombia. Then, we performed numerical simulations according to Runge-Kutta's mathematical method of order 4, where it was possible to know the effects of manipulating the parameters corresponding to the model, and in this way, it is expected to contribute to the design of public health policies in favor of the region.

Based on numerical simulations with city-specific parameters and considering the three different scenarios, it can be given three referent conclusions related to the dynamical behavior about parameters involved in the model. First, the number of sexual partners $c_{h,hw,hm,s}$ and the departure rate in infected individuals α are the most sensitive parameters in the dynamics of infection because infected populations have big changes when these values are slightly modified. Second, scenario 2 shows that the best way to decrease heterosexual contagion in San Juan de Pasto is to increase the departure rate in infected individuals α. Third, scenarios 1 and 3 show that increasing the number of sexual partners in homosexual or heterosexual contact implies an increase in the number of infections.

Thus, the best way to reduce contagion and consequently to reach a disease-free equilibrium is mainly decreasing the number of homosexual partners since they are the most affected population by the virus and the most likely to get infected and to spread the infection. Increasing the departure rate of infected individuals leads to a decrease in infected heterosexual men and infected women, but it is not enough to prevent and curb the rate of contagion.

With the population parameters of San Juan de Pasto, we performed several numerical simulations modifying parameters that return the basic reproduction number greater than or less than one, it suggests that: when $R_{0}^{het}<1$ and $R_{0}^{hom}>1$, there is a general decline in the HIV infection over a period of few years, but the infection persists. Thus, it can be concluded that the most important observation from our findings is that in the population of the city, there is a short-term rise of HIV infection, in which there is a significant increase of new HIV infections followed by a substantial decline in the generation of new infections.

## Notes

^*^
Sensitivity analysis according to the Latin Hypercube Sampling method is demonstrated in the thesis work of Espitia (2021).

## References

[B1] Afassinou, K. 2016. Analysis of multiple control strategies for pre-exposure prophylaxis and post-infection interventions on HIV infection [PhD Thesis]. University of Kwazulu-Natal.

[B2] Akudibillah, G., Pandey, A., and Medlock, J. 2018. Optimal control for HIV treatment. Math. Biosci. Eng. 16, 373–396. 30674124 10.3934/mbe.2019018

[B3] Anderson, R.M., Medley, G.F., May, R.M., et al. 1986. A preliminary study of the transmission dynamics of the human immunodeficiency virus HIV, the causative agent of AIDS. Math. Med. Biol. J. IMA. 3, 229–263. 10.1093/imammb/3.4.229 3453839

[B4] Apenteng, O.O., and Ismail, N.A. 2019. Modeling the impact of migration on HIV persistency in Ghana. Statistics, Optim. Inf. Comput. 7, 55–65.

[B5] Beloqui, J.A. 2008. Relative risk for AIDS between homo/bisexual and heterosexual men. Rev. Saúde Públ. 42,1–6. 10.1590/s0034-89102008005000014 18408826

[B6] Bhunu, C.P. 2015. Assessing the impact of homelessness on HIV/AIDS transmission dynamics. *Cogent Math*. Stat. 2, 1021602.

[B7] Bhunu, C.P., Mhlanga, A.N., and Mushayabasa, S. 2014. Exploring the impact of prostitution on HIV/AIDS transmission. *Int. Sch. Res*. Notices. 2014, 651025. 10.1155/2014/651025 PMC4897385 27471746

[B8] CAC 2019. Fondo Colombiano de Enfermedades de Alto Costo [Colombian Fund for High-Cost Diseases]. Situación del VIH en Colombia 2019. Available at: https://n9.cl/grpu. Accessed March 8, 2020.

[B9] Chan, S.K., Thornton, L.R., Chronister, K.J., et al. 2014. Likely female-to-female sexual transmission of HIV—Texas, 2012. MMWR. Morb. Mortal. Wkly. Rep. 63, 209–212. PMC5779339 24622284

[B10] Choi, S.K., Divsalar, S., Flórez, J., et al. 2020. Estrés, salud y bienestar de las personas LGBT en Colombia, resultados de una encuesta nacional [Stress, health and wellbeing of LGBT people in Colombia, results of a national survey]. Williams Institute, UCLA School of law. Available at: https://n9.cl/cdatr. Accessed March 8, 2021.

[B11] CNPV 2019, Resultados Censo Nacional de Población y Vivienda, Pasto Nariño [Results of the National Census of Population and Housing, Pasto Nariño]. Available at: https://n9.cl/g5wg. Accessed February 23, 2021.

[B12] Cohen, M.S., Chen, Y.Q., McCauley, M., et al. 2011. Prevention of HIV-1 infection with early antiretroviral therapy. N. Engl. J. Med. 365, 493–505. 21767103 10.1056/NEJMoa1105243 PMC3200068

[B13] Cornejo, G., Martínez, J., and Vidal, S. 2020. LGBT studies without LGBT studies: Mapping alternative pathways in Perú and Colombia. J. Homosex. 67, 417–434. 30372382 10.1080/00918369.2018.1534411

[B14] DANE 2019. Departamento Administrativo Nacional de Estadística [National Administrative Department of Statistics]. Available at: https://www.dane.gov.co. Accessed February 23, 2021.

[B15] Del Romero, J., Baza, M.B., Río, I., et al. 2016. Natural conception in HIV-serodiscordant couples with the infected partner in suppressive antiretroviral therapy: A prospective cohort study. Medicine. 95, e4398. 27472733 10.1097/MD.0000000000004398 PMC5265870

[B16] Espitia C. 2021. (in press). Mathematical Modeling of HIV/AIDS Spread in Human Population [Unpublished doctoral dissertation]. University of Campinas. Brazil.

[B17] Fernández, D.Y. 2019. Comportamiento sexual y prevalencia de VIH en HSM en tres ciudades de Colombia Bogotá, Medellin y Cali en 2019 [Sexual behavior and HIV prevalence in MSM in three Colombian cities Bogotá, Medellin and Cali in 2019.]. Availabe at: https://n9.cl/gdo78. Accessed March 8, 2019.

[B18] Glick, S.N., Morris, M., Foxman, B., et al. 2012. A comparison of sexual behavior patterns among men who have sex with men and heterosexual men and women. *J. Acquir. Immune Defic*. Syndr. 60, 83. 10.1097/QAI.0b013e318247925e PMC3334840 22522237

[B19] Holland, J.J. 2007. Notes on R0. Department of Anthropological Sciences. Technical Report 323. Stanford University. Department of Anthropological Sciences, Stanford, CA.

[B20] IDSN 2019. Instituto Departamental de Salud de Nariño, fuente SIVIGILA. Available at: https://n9.cl/g6at5. Accessed March 8, 2021.

[B21] Kaur, N., Ghosh, M., and Bhatia, S.S. 2012. Modeling the spread of HIV in a stage structured population: Effect of awareness. *Int. J*. Biomath. 5, 1250040.

[B22] Kwakwa, H.A., and Ghobrial, M.W. 2003. Female-to-female transmission of human immunodeficiency virus. Clin. Infect. Dis. 36, e40–e41. 12539088 10.1086/345462

[B23] Lloyd, A.L., and May, R.M. 1996. Spatial heterogeneity in epidemic models. J. Theor. Biol. 179, 1–11. 8733427 10.1006/jtbi.1996.0042

[B24] Mastro, T.D., and De Vincenzi, I. 1996. Probabilities of sexual HIV-1 transmission. AIDS. 10(Suppl. A), S75–S82. 10.1097/00002030-199601001-00011 8883613

[B25] Mukandavire, Z., Chiyaka, C., Magombedze, G., et al. 2009. Assessing the effects of homosexuals and bisexuals on the intrinsic dynamics of HIV/AIDS in heterosexual settings. Math. Comput. Model. 49, 1869–1882.

[B26] Nettleman. 2019. HIV Tests, Symptoms, Signs, and Stages of Infection. Available at: https://n9.cl/f5da. Accessed March 13, 2021.

[B27] Omondi, E.O., Mbogo, R.W., and Luboobi, L.S. 2018. Mathematical analysis of sex-structured population model of HIV infection in Kenya. Lett. Biomath. 5, 174–194.

[B28] Omondi, E.O., Mbogo, R.W., and Luboobi, L.S. 2019. A mathematical modeling study of HIV infection in two heterosexual age groups in Kenya. Infect. Dis. Model. 2019, 83–98. 10.1016/j.idm.2019.04.003 PMC6488544 31061932

[B29] Ostadzad, M.H., Shahmorad, S., and Erjaee, G.H. 2016. Study of public health education effect on spread of HIV infection in a density-dependent transmission model. Diff. Equ. Dyn. Syst. 28, 201–215.

[B30] Rosario, M., Schrimshaw, E.W., Hunter, J., et al. 2006. Sexual identity development among lesbian, homosexual, and bisexual youths: Consistency and change over time. J. Sex Res. 43, 46–58. 16817067 10.1080/00224490609552298 PMC3215279

[B31] Simon, B.R., Bockting, W.O., Ross, M.W., et al. 2008. The relationship between homosexuality, internalized homo-negativity, and mental health in men who have sex with men. J. Homosex. 55, 185–203. 18982569 10.1080/00918360802129394

[B32] Sun, X., Xiao, Y., Peng, Z., et al. 2013. Modeling HIV/AIDS epidemic among men who have sex with men in China. BioMed. Res. Int. 2013, 1–18. 10.1155/2013/413260 PMC3806247 24195071

[B33] Thompson, E.M., and Morgan, E. 2008. “Mostly straight” young women: Variations in sexual behavior and identity development. *Dev*. Psychol. 44, 15. 10.1037/0012-1649.44.1.15 18194001

[B34] UNAIDS. 2021. Programa conjunto de las Naciones Unidas sobre el VIH/SIDA, ONUSIDA [Joint United Nations Programme on HIV/AIDS (UNAIDS)]. Available at: https://n9.cl/tx8a. Accessed February 4, 2021.

[B35] Van den Driessche, P. 2017. Reproduction numbers of infectious disease models. Infect. Dis. Model. 2, 288–303. 29928743 10.1016/j.idm.2017.06.002 PMC6002118

[B36] Yang, W., Shu, Z. L am, J., et al. 2017. Global dynamics of an HIV model incorporating senior male clients. Appl. Math. Comput. 311, 203–216.

